# Aggresome–Autophagy Involvement in a Sarcopenic Patient with Rigid Spine Syndrome and a p.C150R Mutation in *FHL1* Gene

**DOI:** 10.3389/fnagi.2014.00215

**Published:** 2014-08-19

**Authors:** Patrizia Sabatelli, Silvia Castagnaro, Francesca Tagliavini, Martina Chrisam, Francesca Sardone, Laurence Demay, Pascale Richard, Spartaco Santi, Nadir M. Maraldi, Luciano Merlini, Marco Sandri, Paolo Bonaldo

**Affiliations:** ^1^Institute of Molecular Genetics, CNR-National Research Council of Italy, Bologna, Italy; ^2^SC Laboratory of Musculoskeletal Cell Biology, Rizzoli Orthopedic Institute, Bologna, Italy; ^3^Department of Molecular Medicine, University of Padova, Padova, Italy; ^4^UF Cardiogénétique et Myogénétique, Service de Biochimie Métabolique, Groupe Hospitalier Pitié-Salpêtrière, Paris, France; ^5^UF Cardiogénétique et Myogénétique, Centre de Génétique, Hôpitaux Universitaires de la Pitié Salpêtrière, Paris, France; ^6^Dulbecco Telethon Institute, Venetian Institute of Molecular Medicine, Padova, Italy; ^7^Department of Biomedical Science, University of Padova, Padova, Italy

**Keywords:** myopathy, sarcopenia, FHL1, autophagy, protein aggregates

## Abstract

The four-and-half LIM domain protein 1 (FHL1) is highly expressed in skeletal and cardiac muscle. Mutations of the *FHL1* gene have been associated with diverse chronic myopathies including reducing body myopathy, rigid spine syndrome (RSS), and Emery–Dreifuss muscular dystrophy. We investigated a family with a mutation (p.C150R) in the second LIM domain of *FHL1*. In this family, a brother and a sister were affected by RSS, and their mother had mild lower limbs weakness. The 34-year-old female had an early and progressive rigidity of the cervical spine and severe respiratory insufficiency. Muscle mass evaluated by DXA was markedly reduced, while fat mass was increased to 40%. CT scan showed an almost complete substitution of muscle by fibro-adipose tissue. Muscle biopsy showed accumulation of FHL1 throughout the cytoplasm and around myonuclei into multiprotein aggregates with aggresome/autophagy features as indicated by ubiquitin, p62, and LC3 labeling. DNA deposits, not associated with nuclear lamina components and histones, were also detected in the aggregates, suggesting nuclear degradation. Ultrastructural analysis showed the presence of dysmorphic nuclei, accumulation of tubulofilamentous and granular material, and perinuclear accumulation of autophagic vacuoles. These data point to involvement of the aggresome–autophagy pathway in the pathophysiological mechanism underlying the muscle pathology of *FHL1* C150R mutation.

## Introduction

Four-and-half LIM domain protein 1 (FHL1) is a cysteine-rich double zinc-finger protein encoded by the *FHL1* gene, localized on chromosome X (Dawid et al., 1995; Kadrmas and Beckerle, 2004). To date, three distinct FHL1 splicing isoforms have been identified (Brown et al., 1999; Morgan and Madgwick, 1999; Ng et al., 2001; Purcell et al., 2004; Johannessen et al., 2006; McGrath et al., 2006). FHL1A, also known as skeletal muscle LIM protein 1, is the full-length protein. FHL1B, or SLIMMER, is composed of the first three LIM domains and contains nuclear localization and export sequences and a RBP-J binding region. FHL1C, or KyoT2, is the shortest isoform, which contains only the first two LIM domains and a RBP-J binding region and interacts with PIAS1 (Taniguchi et al., 1998; Wang et al., 2007).

Four-and-half LIM domain protein 1 is highly expressed in skeletal and cardiac muscles (Lee et al., 1998; Brown et al., 1999; Greene et al., 1999; Morgan and Madgwick, 1999), where it localizes in the myofibrillar sarcomeres and in the sarcolemma (Bertrand et al., 2014). This protein has been demonstrated to be involved in several processes, including cellular architecture (McGrath et al., 2003, 2006), myoblast differentiation (Lee et al., 2012), mechanotransduction (Sheikh et al., 2008), and myofiber size (Cowling et al., 2008). FHL1 binds signaling and cytoskeletal proteins as well as transcription factors, acting as a transcriptional regulator of nuclear factor of activated T cells (NFATc1) to enhance the expression of genes that increase myofiber size (Cowling et al., 2008).

Mutations in the *FHL1* gene are responsible for a number of muscular disorders, which exhibit a broad spectrum of clinical features and disease severity ranging from severe childhood onset to milder adult-onset disorders. The diseases described so far include X-linked myopathy with postural muscle atrophy (XMPMA) (Windpassinger et al., 2008), reducing body myopathy (RBM) (Schessl et al., 2009; Shalaby et al., 2009; Selcen et al., 2011; Schreckenbach et al., 2013), X-linked dominant scapuloperoneal myopathy (Quinzii et al., 2008; Chen et al., 2010), rigid spine syndrome (RSS) (Shalaby et al., 2008), hypertrophic cardiomyopathy (Friedrich et al., 2012), and Emery–Dreifuss muscular dystrophy (Gueneau et al., 2009; Knoblauch et al., 2010). RBM is characterized by the presence of intracellular protein aggregates called “reducing bodies (RBs)” mainly containing mutated FHL1 protein, cytoskeletal and intermediate filament proteins, and components of the unfolded protein response pathway (Liewluck et al., 2007). Although scapuloperoneal myopathy, XMPMA, RSS, hypertrophic cardiomyopathy, and Emery–Dreifuss muscular dystrophy share some overlapping clinical features and muscle pathology with RBM, the involvement of protein aggregation in these disorders remains unclear (Wilding et al., 2014).

Reducing bodies morphologically resemble aggresomes, structures proposed to facilitate the sequestration, and degradation of toxic misfolded proteins. Non-functional, damaged, and/or misfolded proteins are removed from the cell by the ubiquitin proteasome system. However, when the capacity of the proteasome is impaired or overwhelmed, polyubiquitinated misfolded proteins cannot be properly cleared and accumulate into the aggresome (Goldberg, 2003; Kawaguchi et al., 2003). Accumulating evidence suggests that aggresomes are substrates for autophagy. Autophagy is a degradation pathway that mediates bulk clearance of cytosolic proteins and organelles by the lysosome in a highly regulated process involving the coordinated actions of a large number of autophagy-related (Atg) genes. In response to particular stimuli, such as proteasomal dysfunction, an isolation membrane forms and expands to sequester portions of cytoplasm into double membrane structures called autophagosomes. The autophagosomes eventually fuse with lysosomes and their contents are degraded by lysosomal hydrolases. One hypothesis is that aggresomes may concentrate aggregated proteins for more efficient autophagic degradation (Bjorkoy et al., 2005, 2006; Iwata et al., 2005). Recent evidence indicates that aggresome formation is mediated by dynein/dynactin-mediated transport of misfolded proteins to the centrosome and involves several regulators, including the E3 ubiquitin-protein ligase parkin (Olzmann et al., 2008). Aggresome clearance is mediated by ubiquitin-binding proteins such as p62/SQSTM1 (Kirkin et al., 2009), an adaptor protein that decides the fate of protein degradation either through the ubiquitin proteasome system or the autophagy–lysosome pathway (Komatsu et al., 2007; Kirkin et al., 2009; Komatsu and Ichimura, 2010). Here, we report evidence of aggresome and autophagy involvement in the muscle of a sarcopenic patient with RSS and p.C150R mutation in the *FHL1* gene.

## Materials and Methods

### Genotyping

The six coding exons and introns boundaries of FHL1 (NM_001159702) were screened for mutations by PCR on DNA from peripheral lymphocytes using primer pairs with a universal sequence (Table on demand). Exon 5 was sequenced with primers:

$$\begin{array}{l} 5\text{PUF}:\;5\prime -\text{ACCGTTAGTATGCGAGTTGGATTCAGGCACTGGATCCTA}-3\prime \\ 5\text{PUR}:\;5\prime -\text{TCGGATAGTCAGTCGTTTGCTGTCGTGAGGATGGAATG}-3\prime \end{array}$$

Analysis of sequences was done with SeqScape software (Applied Biosystem).

### Muscle biopsy

Peroneal muscle biopsy of the 34-year-old female was performed after written informed consent, and approval was obtained from the Ethics Committee of the Rizzoli Orthopedic Institute. The muscle sample was frozen in melting isopentane and stored in liquid nitrogen.

### Histochemistry and immunohistochemical analysis

Standard histochemical study was performed, and congophilic deposits were identified by Congo red staining (Bioptica) following the manufacturer’s instructions. Cytochrome oxidase activity was assessed by conventional method. Acridine-orange staining was performed as previously reported (Darzynkiewicz, 1994). For double staining with menadione–nitro blue tetrazolium and anti-FHL1 antibodies, 10 μm-thick frozen sections were incubated with menadione-NBT solution in Gomori-Tris-HCl buffer at pH 7.4, without the addition of α-glycerophosphate substrate (Brooke and Neville, 1972), followed by incubation with anti-FHL1 antibody (Aviva System) and TRITC conjugated anti-rabbit secondary antibody (DAKO). Seven micrometer-thick non-fixed frozen sections were incubated with antibodies against laminin α2 chain, collagen VI, parkin (Millipore), desmin, developmental myosin heavy chain (d-MHC), fast myosin heavy chain, dystrophin (DYS1, DYS2, and DYS3 antibodies), emerin, lamin A/C (Novocastra), LC3 (Novus Biologicals), p62 (Progen Biotechnik), pericentrin, α-B-crystallin (Abcam), ubiquitin (Santa Cruz Biotechnologies), and histones (Chemicon), and revealed with FITC or TRITC conjugated anti-rabbit, anti-mouse, or anti-guinea pig secondary antibodies. Samples were stained with DAPI, mounted with anti-fading reagent (Molecular Probes), and observed with a Nikon epifluorescent/light microscope.

### Confocal imaging

The confocal imaging was performed with a A1-R confocal laser scanning microscope (Nikon), equipped with a Nikon 60×, 1.4 NA objective, and with a 488 and 561 nm laser lines to excite FITC (green) and TRITC (red) fluorescence signals. The 3D images were processed by stacking up 20–25 consecutive confocal images with surface shaded reconstruction. No deconvolution was applied to the images.

### Transmission electron microscopy

Muscle biopsy was fixed with 2.5% glutaraldehyde in 0.1 M cacodylate buffer pH 7.4 for 3 h at 4°C, post-fixed with 1% osmium tetroxide in cacodylate buffer for 2 h, dehydrated in an ethanol series, infiltrated with propylene oxide and embedded in Epon 812 resin. Ultrathin sections were stained with uranyl acetate and lead citrate (Reynolds) and examined under a Philips EM400 operating at 100 kV.

### Western blotting

Twenty micrometer-thick frozen sections were cut from the muscle biopsy of a healthy individual and of the proband patient. Sections were taken from two different portions of the patient muscle biopsy (referred as P_a_ and P_b_). Muscle sections were lysed in 150 μl lysis buffer (50 mM Tris, pH 7.5, 150 mM NaCl, 10 mM MgCl_2_, 1 mM EDTA, 10% glycerol, 0.5 mM DTT, 2% sodium dodecyl sulfate, 1% Triton X-100) supplemented with phosphatase inhibitors (Sigma-Aldrich) and protease inhibitors (Roche), heated at 70°C for 10 min and centrifuged at 16,100 g for 10 min at 4°C. The protein content of each lysate was determined by the BCA Protein Assay kit (Pierce) and 30 μg of total proteins were separated by SDS-PAGE (Invitrogen) and immunoblotted as previously described (Chen et al., 2014). Membranes were probed with primary antibodies against FHL1 (ab23937 Abcam), LC3 (Thermo Scientific), p62 (Progen Biotechnik), ubiquitin (Cell Signaling Technologies), beclin 1 (Cell Signaling), BNIP3 (Sigma), vinculin (Sigma), or GAPDH (Millipore). Proteins were revealed with anti-rabbit, -mouse (Bethyl), -goat (Santa Cruz Biotechnologies), or -guinea pig (Sigma) HRP-conjugated secondary antibodies using the ECL reagent (Pierce-Thermo Scientific). Densitometric quantification of protein bands was performed by the ImageJ software (US National Institute of Health). Western blotting and quantifications are representative of at least three independent experiments.

## Results

**Figure 1:**
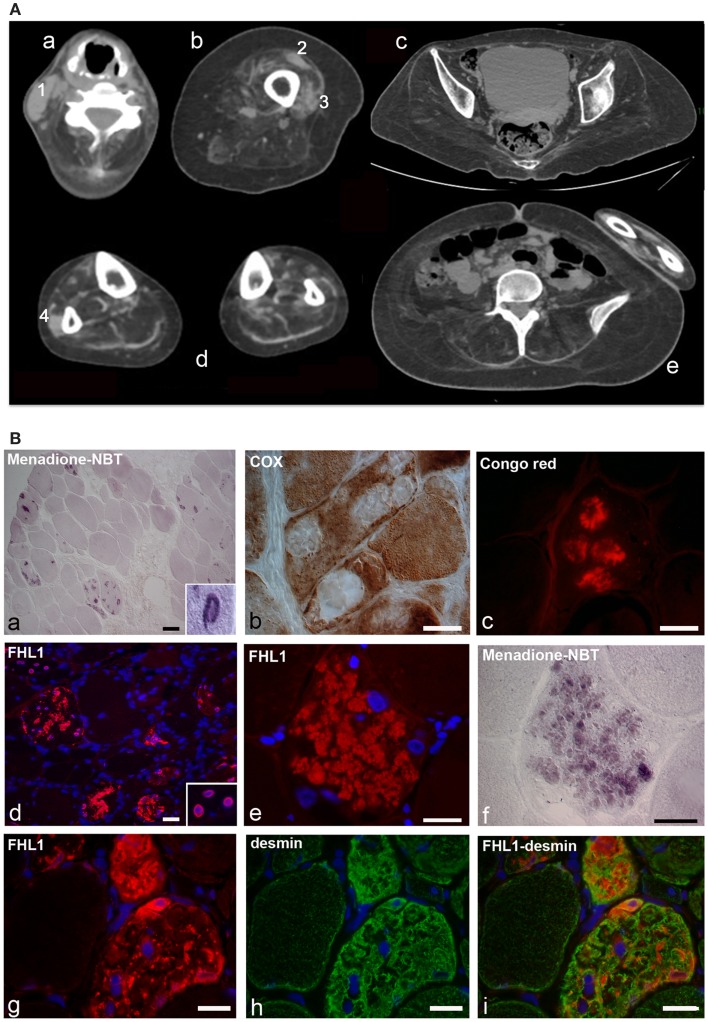
**(A)** Patient with FHL1 mutation: index case. Muscle CT imaging of the neck (a), left thigh (b), pelvis (c), lower legs (d), and abdominal (e) muscles. In the neck, there is a relative preservation of the left sternocleidomastoideus (1) and almost complete involvement of all the other muscles (a). In the left thigh, only the rectus femoris (2) and vastus lateralis (3) are relatively spared (b). In the pelvis (c) and in the abdomen (e), there is a marked degeneration of all muscles. In the lower legs, only the peroneus (4) of the right leg is relatively spared (d). **(B)** Histochemistry and immunohistochemical analysis of patient muscle biopsy. Cross-sections show the presence of RBs in several muscle fibers, as revealed by menadione-NBT staining (a). Granular deposits are detectable at the nuclear rim of some myonuclei (a, inset). RBs appear devoid of oxidative activity (cytochrome oxidase, COX in b) and display affinity to Congo red staining (c). Immunofluorescence analysis of FHL1 shows protein accumulation throughout the cytoplasm of several muscle fibers (d) and around myonuclei (d, inset). FHL1 immunolabeling (e), followed by menadione-NBT staining (f), demonstrates that FHL1 accumulates in RBs (f). Double labeling for FHL1 and desmin (g–i) shows a marked increase of desmin in FHL1-accumulating myofibers. Scale bar, 20 μm.

The proband is a 34-year-old woman who noticed the inability to extend the right thumb at age 20. Soon after, she manifested neck weakness and limitation of flexion. Progression of weakness was rapid and she started to have difficulty in climbing stairs and getting up from the floor. At age 24, she started falling several times while walking. At age 26, the patient lost ambulation and was wheelchair bound. Examination at age 34 showed an atrophic phenotype with marked diffuse muscle wasting and weakness and prominent contractures. She had normal facial muscle strength, a minimal residual motor function in the elbow extensors and in the right biceps, but was profoundly weak in all the other muscles. She revealed marked contractures involving proximal and distal joints. The most striking contractures were in the neck muscles causing a fixed hyperextended neck that was also impossible to move in any direction. She showed a progressive decline in the respiratory function with a forced vital capacity 59% of predicted at age 25, 45% at age 27, and 13% at age 34. She refused to undergo mechanical ventilation. Cardiac investigation, including echocardiography and Holter, revealed no cardiac involvement. Muscle CT showed that all muscles were atrophic and substituted by fat and connective tissue, including the axial muscles, with a minimal sparing of the rectus femoris and vastus lateralis on the left, and of the peroneus on the right (Figure 1A). She was underweight (BMI = 17.1). However, according to her body composition, as revealed by DXA, she was sarcopenic/obese (appendicular lean body mass index of 3.27 kg/m^2^ and total body fat of 44.4%) (Baumgartner et al., 2004). Her brother had a similar atrophic phenotype with marked rigidity of the spine and diffuse contractures but with more rapid progression, as he lost ambulation at age 18 and underwent tracheostomy at age 28. Their mother at age 58 had a mild lower limbs weakness and no contractures.

Sequencing of the *FHL1* gene in the index case identified a single missense mutation c.448T > C in exon 5 resulting in the replacement at codon 150 of a cysteine residue with an arginine residue (p.Cys150Arg). Nucleotide c.448 and residue p.150 of the *FHL1* gene are highly conserved among species and evolution, and all prediction softwares conclude for a pathogenic mutation. Analysis of the family showed that her brother and mother also have the same *FHL1* mutation.

Muscle biopsy showed fiber size variability, numerous internal nuclei, and increased endomysial and perimysial tissue. Several muscle fibers showed menadione-NBT positive aggregates, consistent with the presence of RBs (Figure 1B). RBs were devoid of oxidative enzyme activity and displayed an intense congophilia, indicative of the presence of amyloid deposits. Immunohistochemistry with FHL1 antibody revealed the presence of protein aggregates in about 20% of muscle fibers. Consistent with previous reports (Selcen et al., 2011; Feldkirchner et al., 2013; Bertrand et al., 2014), FHL1 deposits were detected throughout the cytoplasm and around myonuclei. Double staining with menadione-NBT revealed that FHL1 deposits strongly correlated with RBs, although with a more diffuse pattern. Desmin and α-B-crystallin (not shown) were strongly up-regulated in affected muscle fibers but they did not co-localize with FHL1 deposits (Figure 1 and data not shown). Sarcolemmal components, such as dystrophin and laminin α2, were not detected in RBs; collagen VI was increased in the endomysium and perimysium, possibly as a consequence of active fibrosis (data not shown).

**Figure 2:**
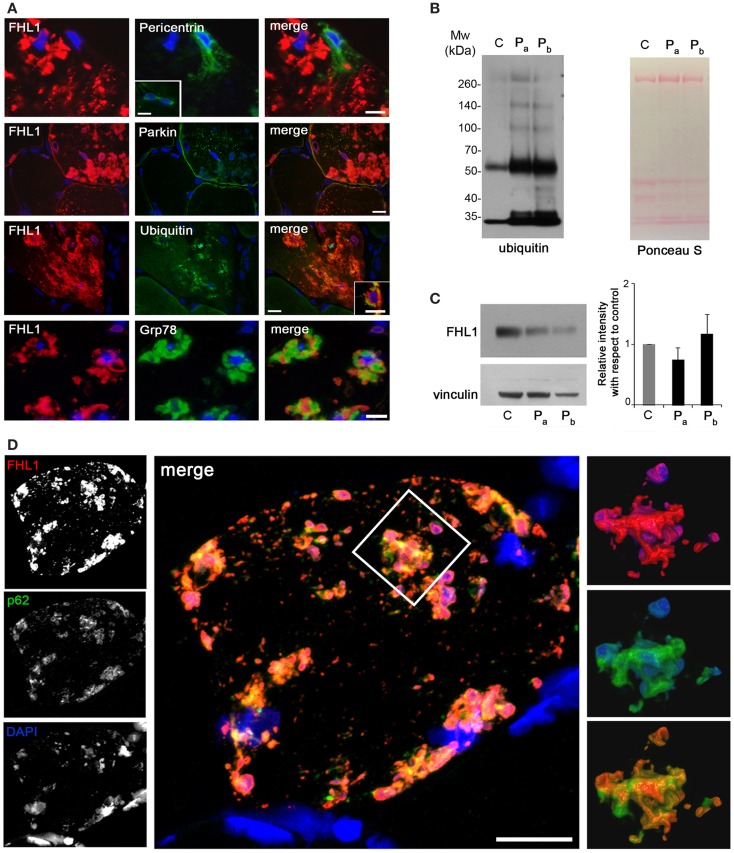
**(A)** Double labeling of FHL1 with pericentrin, parkin, ubiquitin, and Grp78 in cross-sections of patient muscle biopsy. Scale bar, 20 μm. **(B)** Western blot analysis for ubiquitin in total protein extracts from muscle biopsies derived from a healthy donor **(C)** and from the patient (two different fragments of the index case biopsy, P_a_ and P_b_). Increased reactivity for ubiquitin is clearly detectable in patient extracts. Ponceau S staining is shown as loading control. **(C)** Western blot analysis for FHL1 and vinculin in total protein extracts from muscle biopsies derived from a healthy donor **(C)** and from the index case (P_a_ and P_b_). Quantification of the FHL1 protein level showing the relative western blot intensity with respect to control. Densitometric quantification was performed by three independent western blot experiments (*P* > 0.05, P_a_ and P_b_ vs C). **(D)** Confocal immunofluorescence imaging of the patient muscle biopsy labeled with FHL1 antibody (red), p62 antibody (green), and DAPI (blue). The maximum intensity projections of the single channels (white) are shown on the left, the merged image of confocal projections are shown in the middle, and the 3D surface shaded reconstruction of an enlargement of the area defined by the white box is shown on the right. Scale bar, 10 μm.

Reducing bodies displayed aggresome features as indicated by association with ubiquitin and with the luminal endoplasmic reticulum chaperone Grp78, in agreement with a previous work (Wilding et al., 2014). Consistent with aggresome formation, parkin, an E3 ubiquitin ligase involved in retrograde transport of misfolded proteins to centrosome (Garcia-Mata et al., 1999), and pericentrin, a marker of centrosome, were increased in affected myofibers (Figure 2A). Western blot analysis showed a patient-specific increase of ubiquitin (Figure 2B), confirming the massive presence of aberrant ubiquitinated proteins. Western blotting for FHL1 in the soluble fraction of patient muscle biopsy showed no significant change of FHL1 protein levels with respect to the control (Figure 2C).

**Figure 3:**
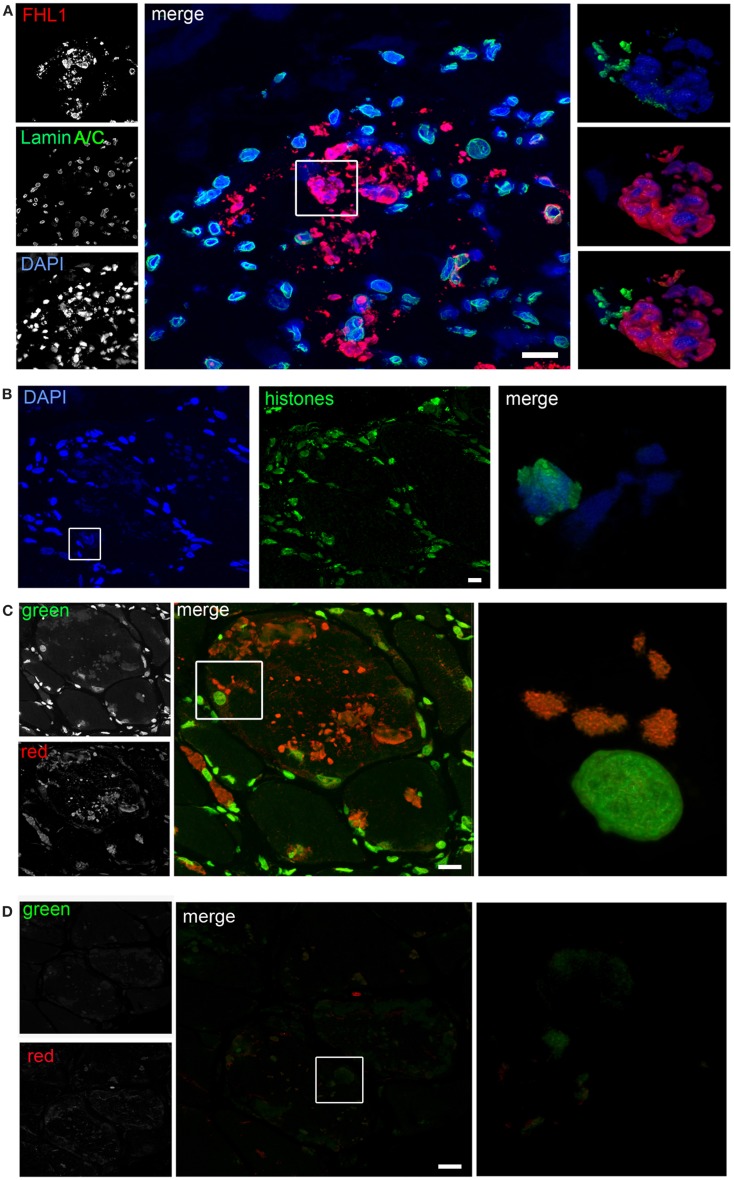
**(A)** Confocal immunofluorescence imaging of the patient muscle biopsy labeled with FHL1 antibody (red), lamin A/C antibody (green), and DAPI (blue). The maximum intensity projections of red and green channels (white) are shown on the left, the merged image of confocal projections are shown in the middle, and the 3D surface shaded reconstruction of an enlargement of the area defined by the white box is shown on the right. **(B)** Confocal immunofluorescence imaging of the patient muscle biopsy labeled with an anti-histones antibody (green) and DAPI (blue), together with 3D surface shaded reconstruction (merge). **(C)** Acridine-orange staining. The maximum intensity projections of the single channels (white) are shown on the left, the merged image of confocal projections are shown in the middle, and the 3D surface shaded reconstruction of an enlargement of the area defined by the white box is shown on the right. **(D)** Acridine-orange staining after DNase digestion on frozen sections of the patient’s muscle biopsy showing that the treatment completely removed nuclear and RBs associated DNA. The maximum intensity projections of the red and green channels (white) are shown on the left, the merged image of confocal projections are shown in the middle, and the 3D surface shaded reconstruction of the area defined by box is shown on the right. Scale bar, 10 μm.

Confocal imaging revealed a clear co-localization of FHL1 with p62 labeling (Figure 2D). FHL1/p62-positive aggregates also stained with DAPI, indicating the presence of nuclear material. Interestingly, DAPI-positive structures were not surrounded by nuclear lamina, as indicated by the absence of lamin A/C (Figure 3A) and emerin (not shown). In addition, DAPI-positive structures did not associate with histones (Figure 3B) and displayed an intense red fluorescence when stained with acridine orange (Figure 3C), a metachromatic dye that differentially stains double-stranded DNA and single-stranded DNA or RNA. Notably, DNase treatment strongly reduced the acridine-orange staining (Figure 3D). Altogether, these data suggest that FHL1/p62 aggregates also include single-stranded DNA, possibly due to nuclear degradation.

**Figure 4:**
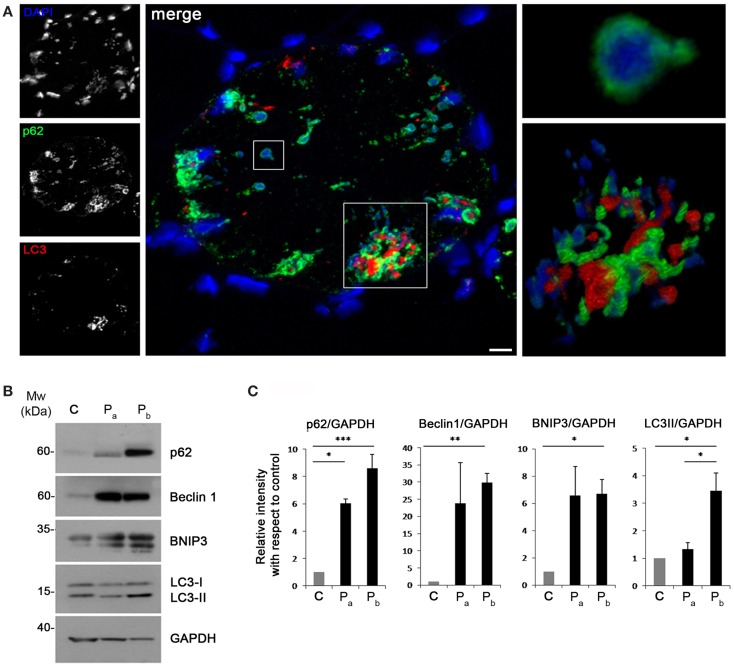
**(A)** Confocal immunofluorescence imaging of the patient muscle biopsy labeled with LC3 antibody (red), p62 antibody (green), and DAPI (blue). The maximum intensity projections of the single channels (white) are shown on the left, the merged image of confocal projections are shown in the middle, and the 3D surface shaded reconstruction of an enlargement of the areas defined by the white boxes are shown on the right. Scale bar, 10 μm. **(B)** Western blot analysis for the autophagic markers LC3, Beclin 1, BNIP3, and p62 in muscle biopsies derived from a healthy donor **(C)** and from the index case (P_a_ and P_b_). GAPDH was used as a loading control. **(C)** Quantification of the protein levels showing the relative western blot intensity with respect to control. Densitometric quantification was performed by three independent western blot experiments (***P < 0.001; **P < 0.01; *P < 0.05).

Recent studies have suggested that aggresomes are substrates for autophagy (Yao, 2010). LC3 immunolabeling on the patient muscle biopsy revealed the presence of autophagosomes in proximity of p62 aggregates (Figure 4A), suggesting the involvement of the autophagic pathway in aggresome clearance. Moreover, analysis of the protein levels of several autophagic markers showed a strong accumulation of p62, confirming the presence of an elevated number of aggresomes (Figures 4B,C). Notably, Beclin 1 and BNIP3, two well-known positive regulators of autophagy, were strongly increased in the patient biopsy, indicating that autophagy induction is taking place, likely in response to the need of clearing the accumulating aggregates. This was further confirmed by the slight increase of LC3 lipidation observed in the patient biopsy (Figures 4B,C). All together, these data indicate that autophagy is strongly induced in the muscle biopsy from the patient.

**Figure 5:**
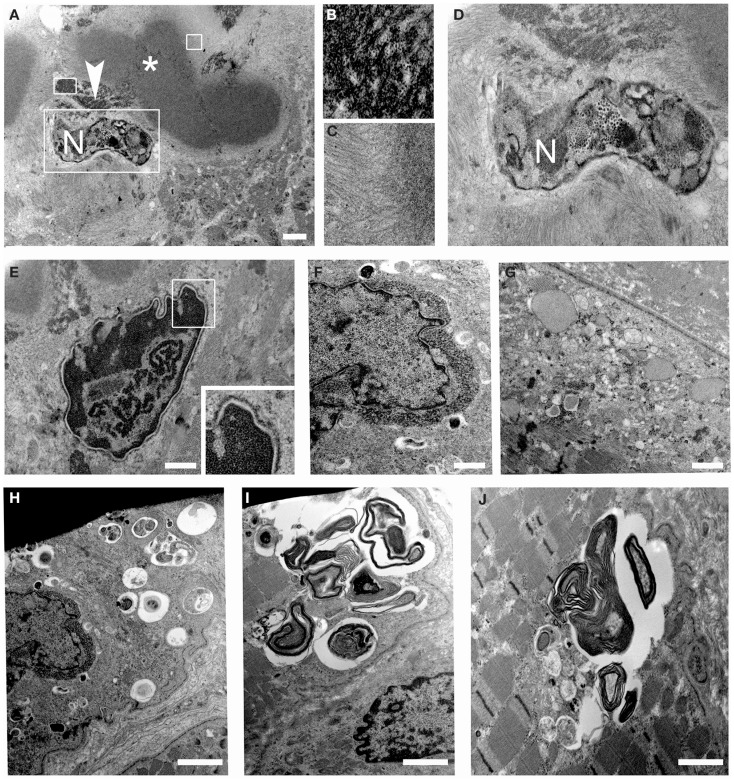
**Transmission electron microscopy of patient muscle biopsy**. **(A–D)** Images showing cytoplasmic body (asterisk) and tubulofilamentous aggregates (arrowhead) close to degenerated nuclei (N). Higher magnification of the areas defined by boxes in **(A)** is shown in **(B–D)**. **(E,F)** Images showing altered nuclei with granular material accumulated at the nuclear rim. **(G)** Images showing dilated sarcoplasmic reticulum filled with amorphous material in a myofiber. **(H–J)** Images showing autophagic vacuoles and myelin figures close to nuclei and throughout some sarcomeres of muscle fibers. Scale bar, 500 nm.

Ultrastructural analysis showed cytoplasmic bodies and tubulofilamentous material associated with nuclear alterations and autophagic vacuoles (Figure 5). Tubulofilamentous aggregates ranged from 14 to 1.2 nm. Dysmorphic nuclei showed condensed heterochromatin, ribonucleoprotein aggregates, enlarged nucleoli, and condensed granular material at the outer face of the nuclear cisterna. In addition, a reduced number of nuclear pores were also detected in nuclei with hypercondensed heterochromatin. Autophagic vacuoles, and in particular autophagolysosomes as indicated by the presence of a single membrane, were frequently found in proximity of altered nuclei. Other inclusions consisted of myelinic bodies and aggregates of sarcoplasmic reticulum.

## Discussion

In this work, we provided data showing that aggresome and autophagy are involved in the pathophysiological defects underlying the muscle pathology of a sarcopenic patient with RSS and carrying a *FHL1* p.C150R mutation.

In our family, the female index case and her brother had a typical RSS (Moghadaszadeh et al., 2001). The p.C150R mutation has already been reported in patients with RSS (Schessl et al., 2010; Selcen et al., 2011). Interestingly, another female patient (Selcen et al., 2011) presented extensor pollicis weakness, which was the first symptom noticed by our index case. Our patient had an atrophic phenotype. Underweight by body mass index, she was recognized to be sarcopenic-obese as determined by DXA given the marked reduction of lean body mass with relative increase of fat mass (Baumgartner et al., 2004). The sarcopenic condition was also reflected in the muscle CT that showed diffuse end stage degeneration. The brother of the index case had a severe progressive course; he lost ambulation at age 18, and underwent tracheostomy at age 28. In previously reported families, male patients were the most affected, while female carriers showed varying manifestations usually mild and some were asymptomatic (Schessl et al., 2010; Selcen et al., 2011). In our family, the two female patients had a very different course: severe and progressive in the index case and mild in her mother who was ambulant and without spine rigidity at age 58. Because of X chromosome inactivation, heterozygous women are mosaic for X-linked gene expression. This may explain the much milder phenotype in the mother compared with that of her daughter (Schessl et al., 2010; Selcen et al., 2011). The less affected mother was also much less atrophic, pointing to a possible differential activation of muscle atrophy pathways. However, no muscle biopsy of the mother was available, and X-inactivation studies were not performed in this family.

Muscle findings included menadione-NBT-positive aggregates, consistent with RBs, which also contained FHL1. The same *FHL1* mutation was previously reported in a family with RBM phenotype (Schessl et al., 2010) and in two patients with RBs and myofibrillar myopathy (Selcen et al., 2011). Also in those patients, the mutated FHL1 protein accumulated in RBs, pointing to a causative effect of this mutation in RB formation. The mutated cysteine residue localizes in the second LIM domain of the protein and it is expected to affect all FHL1 isoforms, i.e., full-length FHL1A and the shorter FHL1B and FHL1C polypeptides. Cys150 is a crucial coordinating residue in the second LIM domain (Michelsen et al., 1994) and mutations occurring at this site are predicted to induce protein misfolding. It has been proposed that the accumulation of misfolded FHL1 polypeptides results in the characteristic RB aggregates observed in muscle of RBM patients as well as in C2C12 myoblasts transfected with the mutant FHL1 protein (Schessl et al., 2008).

Aggresomes are structures proposed to facilitate the sequestration and degradation of toxic misfolded proteins. In agreement with previous reports (Bertrand et al., 2014), the RBs of the proband displayed characteristics of aggresomes, as indicated by the increase of proteins involved in aggresome formation and by the accumulation of ubiquitin, Grp78, p62, and cytoskeletal components, such as desmin and α-B-crystallin. Moreover, the FHL1-containing aggresomes were mainly accumulated around nuclei. It is well known that aggresome formation is mediated by the dynein/dynactin-mediated transport of misfolded proteins to the centrosome, as confirmed by the presence of aggresomes in the perinuclear region and matching with centrosome markers (Olzmann et al., 2008). In muscle cells, the centrosome undergoes redistribution at the nuclear rim during differentiation (Bugnard et al., 2005). This pattern persists in adult muscle (Srsen et al., 2009), as indicated by the localization of centrosome markers on the outer membrane of the nuclear cisterna. This peculiar positioning of the centrosome at the nuclear rim of muscle cells accounts for the recruitment of granular material with aggresome-like features we observed in nuclei of the *FHL1* mutated muscle fibers. In addition, we found that nuclei with perinuclear granular material appeared dysmorphic, with dramatic changes of the nuclear envelope and hypercondensed heterochromatin. These data, in addition to the finding of single-stranded DNA in aggresomes, suggest that the aggresome accumulation at the nuclear rim may induce nuclear degradation. This hypothesis is consistent with the alterations of the nuclear envelope in cells containing inclusion bodies that were described in patients affected by Huntington disease and in transgenic mice expressing mutant huntingtin (Waelter et al., 2001).

We also found that Grp78, an endoplasmic reticulum chaperone up-regulated during the unfolded protein response, was strongly increased and associated to the FHL1 deposits in the proband muscle biopsy. This finding is in agreement with previous work showing increased expression of Grp78 and unfolded protein response in patients with RBM (Liewluck et al., 2007). However, the association of Grp78 with aggresomes may be also due to retrograde transport from the endoplasmic reticulum, as hypothesized by the formation of aggresome-like inclusion bodies induced by mutant huntingtin (Garcia-Mata et al., 2002).

The association of FHL1 with p62 we detected in the proband muscle biopsy indicates that the mutant FHL1 protein is targeted to degradative pathways. p62 is a multifunctional protein containing a number of protein–protein interaction motifs that are involved in protein aggregation and degradation (Moscat and Diaz-Meco, 2009a,b). It has been hypothesized that p62 may act as a critical ubiquitin chain-targeting factor that shuttles substrates for proteasomal degradation (Seibenhener et al., 2004). On the other hand, a role for p62 in aggregate formation for autophagic degradation was also hypothesized (Komatsu et al., 2007; Kirkin et al., 2009; Komatsu and Ichimura, 2010). The strong increase of Beclin 1 and BNIP3 levels we detected in the patient biopsy indicates that autophagy induction is taking place, likely to help the clearance of accumulating aggregates. This is further confirmed by the slight increase of LC3 lipidation and by the accumulation of LC3 deposits in proximity to aggresomes in the proband muscle. The presence of autophagic vacuoles and myelin figures further confirms the involvement of the autophagic pathway in the pathophysiological alterations of this patient. Interestingly, the presence of autophagosomes and autophagic vacuoles was also reported in muscle biopsies of RBM patients (Bertrand et al., 2014). It is also interesting to consider that FHL1 null mice, lacking global FHL1 expression and without aggregates accumulation, display susceptibility to autophagy, as indicated by increased LC3 lipidation in skeletal muscle (Domenighetti et al., 2014). These findings point to a causative role of FHL1 deficiency in autophagy activation, and indicate that activation of the autophagic pathway in FHL1-related myopathies may be a common pathophysiological mechanism, independent from the accumulation of protein aggregates. Although future studies of the autophagic flux in muscle cells from patients and animal models for FHL1 deficiency are needed in order to understand in detail how and to which extent deregulation of autophagy contributes to the pathogenesis of FHL1-related myopathies, our data demonstrate for the first time the coexistence of aggresomes and autophagy in the muscle biopsy of a patient with severe sarcopenia caused by p.C150R mutation in *FHL1*. These findings add new insights in delineating the altered mechanisms involved in the pathogenesis of *FHL1*-associated diseases.

## Conflict of Interest Statement

The authors declare that the research was conducted in the absence of any commercial or financial relationships that could be construed as a potential conflict of interest.
